# Mapping the evidence for monitoring fluoride exposure in community prevention programmes for oral health using nail clippings and spot urine samples: a scoping review

**DOI:** 10.1186/s12903-022-02615-2

**Published:** 2022-12-08

**Authors:** Elizabeth Adjoa Kumah, Fatemeh Eskandari, Liane B. Azevedo, Sherley John, Fatemeh Vida Zohoori

**Affiliations:** 1 Department of International Public Health, Liverpool School of Tropical Medicine, Liverpool, UK; 2 School of Health and Life Sciences, Teesside University, Middlesbrough, UK; 3 School of Human and Health Sciences, University of Huddersfield, Huddersfield, UK

**Keywords:** Fluoride, Dental caries, Spot urine, Nail, Biomarker

## Abstract

**Background:**

There is an increased interest in identifying practical and accurate biomarkers for fluoride exposure. Due to the narrow ‘dose-gap’ between the benefit of caries reduction and the risk of dental fluorosis, monitoring of fluoride exposure is vital when introducing any fluoridation programme for the prevention of dental caries. This scoping review aimed to ascertain the nature and extent of the available evidence on how spot urine and nail clippings are used to measure fluoride intake/exposure, by using a unique approach of mapping the studies according to population, setting, type of study design, methodology and analytical approach in community prevention programmes.

**Methods:**

Multiple relevant databases were searched up to July 2021 for any study designs, including randomised controlled studies, quasi-experimental studies, surveys, retrospective and prospective cohort studies, case studies, phenomenological studies, and expert opinions.

**Results:**

The search retrieved 9,222 studies of which 155 met the inclusion criteria. A high proportion of the studies (25.2%) originated from Latin America and the Caribbean continent subregion. However, per country, China recorded the highest number, followed by India and Mexico. The majority (62.6%) employed a cross-sectional study design, and 65.8% combined participants from different age groups. Of the included studies, 82.6% used spot urine samples as a biomarker for assessing fluoride intake/exposure. Water fluoride concentration was reported in 66.5% of the studies with 46.6% of all included studies reporting a water fluoride concentration of > 1.2 mg/L. The methods used in assessing oral hygiene and dietary intake were not reported in 72.3% and 71.0% of the included studies, respectively. Only 35.5% of the included studies assessed the relationship between fluoride exposure and excretion.

**Conclusions:**

This review revealed a large variability in the way in which spot urine samples and/or nail clippings are used to measure fluoride exposure in different settings and situations. Particularly, there are inconsistencies in the methodologies and the analytical approaches used in assessing fluoride exposure. Therefore, there is a need for more rigorous primary research studies using standardised approaches to determine the suitability of spot urine samples and nail clipping as biomarkers for monitoring fluoride exposure.

**Supplementary Information:**

The online version contains supplementary material available at 10.1186/s12903-022-02615-2.

## Introduction

Dental caries, a preventable condition, remains an important global public health problem. The use of fluorides for population-based prevention of dental caries has been officially acknowledged by the World Health Organisation (WHO) since the late 1960s [1]. The goals of community-based public health programmes are generally to provide regular, low-level exposure to fluoride in the community through appropriate means such as fluoridated water, salt, milk and fluoride toothpaste. However, excessive chronic exposure to multiple sources of fluoride during the first 6–8 years of life (i.e. when dental enamel is forming) may cause dental fluorosis. Vast majority of dental fluorosis cases, in areas with optimal fluoride (0.7–1.0 mg/L) in drinking water, are very mild or mild types, which are not easily noticeable to the affected individuals but requiring a trained specialist to detect [2, 3]. Diet, including water, and unintentional ingestion of fluoridated dental products are the main sources of fluoride exposure.

Considering the narrow ‘dose-gap’ between the benefit of caries reduction and the risk of dental fluorosis, public health authorities should monitor total fluoride exposure of the population before and after introducing any fluoridation or supplementation programme for prevention of dental caries. Direct assessment of total fluoride exposure in a population can be difficult and expensive. Identification of practical and accurate biomarkers for fluoride exposure has therefore gained considerable attention over the recent decades. Biomarkers can be used to detect and prevent diseases through the identification of change in samples or biological systems [4]. They are defined as biochemical, molecular or cellular changes that can be measured in biological media (for example, cells, fluids, or human tissues) and can be used to indicate exposures to environmental chemicals, including fluoride [4].

Daily (24 h) urinary fluoride excretion has been suggested as a suitable biomarker for predicting fluoride intake for groups of people. In its recent publication, the World Health Organisation (WHO) [5] has endorsed the use of daily urinary fluoride excretion for monitoring fluoride exposure in community prevention programmes for oral health. Due to practical difficulties in collecting 24 h urine samples from children, spot urine samples and nail clippings have been suggested [6, 7] as simpler alternatives. However, a clearer perspective is needed to better understand the association between fluoride exposure and either biomarker (spot urine and nail clippings). Likewise, there is a need to map situations including populations, settings and methods as they are strongly associated with intake. Previous reviews in this area have focused on synthesising the evidence on the use of 24 h urine as a biomarker for monitoring fluoride exposure [8] or explored the relationship between various fluoride biomarkers and the severity of dental fluorosis [9]. This scoping review is unique as it adopts a comprehensive systematic scoping approach to synthesise the evidence on monitoring fluoride exposure in community prevention programmes for oral health through the specific use of spot urine and/or nail clippings as biomarkers.

## Aim and objective

The aim of this scoping review was to map the evidence on the use of spot urine samples and nail clippings for monitoring fluoride exposure at a community level. The specific objective was to identify the nature and extent of the available evidence on how spot urine and nail clippings are used to measure fluoride exposure according to the study population, setting, type of study design, methodology and analytical approach. The evidence presented in this article clarifies the extent of research that is available, as well as serving as a guide to future research and policy to inform practice.

## Research question

This review was guided by the following research questions:

What is the available evidence on measuring fluoride exposure using spot urine and nail clippings as biomarkers?

How does this evidence vary according to study population, setting, type of study design, methodology and analytical approach?

## Methods

This scoping review was conducted and reported in accordance with the Joanna Briggs Institute Reviewers Manual [10]. The literature screening process was summarised using the PRISMA statement for reporting of items for systematic reviews and meta-analysis [11]. A comprehensive protocol of this scoping review has been registered in the Open Science Framework (Registration https://doi.org/10.17605/OSF.IO/DSJCY). The selection criteria and methods of analysis were specified prior to commencing the review process.

### Inclusion criteria

#### Types of participants

We considered studies that assessed the use of spot urine and/or nail clippings to monitor fluoride exposure among human participants. Human participants included children and/or adults of any age, gender, or ethnicity.

#### Concept

Studies that have examined fluoride exposure through the use of nail clippings and/or spot urine biomarkers, in terms of study population, setting, type of study design, methodology, and analytical approach. Specifically, the following studies were considered for inclusion: (a) studies that performed independent assessment of either nail clippings or spot urine biomarkers, (b) studies that compared these biomarkers with a different type of biomarker, or with each other.

#### Context

Studies from any geographical location across the globe aimed at assessing fluoride exposure using nail clippings and/or spot urine were considered for inclusion. We also considered studies from any setting, including nurseries, schools, preschools, kindergartens, childcare centres, hospitals, or communities. No date restriction was applied to the search.

#### Study types

We included all original primary research (both quantitative and qualitative) studies, including, but not limited to, randomised controlled studies, quasi-experimental studies, surveys, retrospective and prospective cohort studies, case studies, phenomenological studies, and expert opinions.

### Exclusion criteria

The following exclusion criteria were applied to the title and abstract as well as the full-text review stage.

- Irrelevant problem/focus: studies that did not examine fluoride exposure.
- Irrelevant biomarker: studies that measured exposure to fluorides through biomarkers other than nail clippings and/or spot urine.
- Irrelevant participants: studies that examined fluoride exposure using biomarkers from animal species.
- Irrelevant type of study: review reports, expert opinions and statements on fluoride exposure.
- Irrelevant data output: studies that did not report on the methodologies used for monitoring fluoride exposure, such as the population, setting, type of study design, method, and analytical approach.
- Language: studies reported in a language other than English were excluded due to lack of appropriate translators.

## Search strategy

The search strategy was developed by experienced review authors (EAK and FE), with the aim to find both published and unpublished studies on the subject matter. Search terms included a combination of key concepts in the research question, such as fluoride exposure, fluoride intake, fluoride biomarkers, spot urine, and nail clippings. The Boolean operators ‘AND’ and ‘OR’ were used as follows:

(fluoride intake OR fluoride ingestion OR fluoride dose OR fluoride exposure OR fluoride content OR fluorida* OR fluoride biomarker*) **OR** (groundwater OR consumption OR dose* OR intake OR ingest* OR expos* OR fluorid* content OR fluoridat* OR water OR drinking water OR exp mineral water* OR exp water supply) **OR** (diet* OR supplement* OR dentifrice* OR tablet OR salt OR milk OR dental product* OR fluoride varnish* OR mouth rinse* OR infant milk formula OR food* OR beverage OR fluorid* water* OR drink*) **AND** (spot urin* fluoride concentration* OR spot urin* fluoride excretion OR spot urin* fluoride level* OR spot urin* fluoride retention OR renal fluoride excretion OR spot urin* fluoride OR spot urin* fluoride monitor* OR spot urin* fluoride content OR fluoride balance*) **OR** (nail* OR nail clipping*) **AND** (human* populati* OR adult* OR child* OR wom#n OR female OR adult wom#n OR m#n OR male* OR infant* OR newborn OR neonate OR bab* OR toddler* OR preschooler* OR early childhood).

Relevant databases as well as search engines were searched for eligible papers. The databases searched included Medline, CINAHL, Web of Science, Scopus, ScienceDirect, Sage Journals Online, Campbell Collaboration, Cochrane Collaboration, and Embase. Also, Google and Google Scholar search engines were searched for relevant literature. Furthermore, the following relevant grey literature databases, were searched: OpenGrey, NICE Evidence Search, the Grey Literature Report, Bielefeld Academic Search Engine (BASE), and Australian Bureau of Statistics (ABS). In addition, the reference lists of all included studies were searched to identify additional studies. We also emailed leading experts and relevant researchers to ask for unpublished papers and/or data on the use of spot urine samples and nail clippings for monitoring fluoride exposure. The search for eligible papers began on 20th May 2021 and was completed on 20th June 2021. The detailed search strategy is presented as Additional file 1.

## Management of references

The full set of search results were imported into an Endnote X9 library. Where this was not possible, search results were entered manually into the Endnote Library. The search results were then exported from Endnote into Covidence (a web-based software platform that streamlines the production of systematic/scoping reviews) for screening. We also checked for duplicates with Covidence software to remove those that were not identified by the Endnote library.

## Selection of studies

A two-stage screening process was used to evaluate search results for relevant studies. The first level of screening was done by two independent reviewers (EAK and FE) and involved screening of only titles and abstracts. Subsequently, the full texts of potentially relevant studies were examined independently by those reviewers. Discrepancies between reviewers were resolved through discussions. Where disagreements persisted, a third reviewer (FVZ or LBA) was consulted.

## Data charting

The review authors developed a standardised data extraction form in the Covidence software to aid in extracting relevant information from included studies. Specifically, the data extraction form was designed to collect the following information: year of publication, title, aim/objective of study, study design, country, setting, number of participants, age, gender, exposure/intake data, methods of data collection, analytical procedures, and outcome(s).

The developed data extraction form was pilot-tested using 10% of the included articles, prior to commencing the actual data extraction. Data extraction was undertaken by one reviewer (FE or SJ) and verified by another (EAK), using the Covidence software.

## Data synthesis

Firstly, the extracted data were exported from Covidence into Microsoft Excel for editing and to check for accuracy. Then, the edited data were exported from Excel into SPSS (version 26) for data synthesis. The characteristics of included studies as well as their outcome measures were reported using descriptive statistics.

1. **Characteristics of included studies**

- *Year of publication*: we categorised the ‘year of publication’ of the included studies into three groups: a) studies published before the year 2000, b) studies published between 2000–2013, and c) studies published after 2014. This classification was guided by the World Health Organisation’s manual on monitoring fluoride excretion in community prevention programmes, which was initially released in the year 1999 [12], and the updated version released in 2014 [5].
- *Country in which study was conducted*: we categorised this into six regions using the World Bank Group [13] classifications (i.e., East Asia and Pacific, Europe and Central Asia, Latin America and Caribbean, Middle East and North Africa, North America, South Asia, and Sub-Saharan Africa). The rationale for this was to enable us to assess the regional/geographical distribution of the literature on use of spot urine samples and/or nail clippings as biomarkers for examining fluoride exposure.
- *Study design*: cross-sectional studies, randomised controlled trial, non-randomised controlled trial, cohort study, experimental study, case control study, longitudinal study, qualitative research, and before and after intervention study.
- *Gender*: male, female, both
- *Age*: 0–2 years, 3–4 years, 5–6 years, 7–12 years, 13–18 years, and > 18 years.
- *Study Setting*: nursery, schools, preschools, kindergartens, childcare centres, hospitals, industries, and community settings.

2. **Outcome measures**

- Type of biomarker: toenail/fingernail clippings, spot urine, or other
- Source of fluoride exposure: water, dental products, diet, fluoride supplements (tablet), fluoridated salt, fluoridated milk, other or not reported
- Reporting of water fluoride concentration of the area (i.e., the study setting/community): yes, no
- Dietary intake assessment method: 24 h dietary recall, diet history, duplicate method, food diary, food frequency, household survey, observed food frequency, other or not reported
- Oral hygiene assessment method: applied/expectorated toothpaste, questionnaire, other or not reported.
- Validity of dietary intake assessment methods: yes, no or not reported
- Reporting of daily (24 h) urinary fluoride excretion: yes, no
- Method of validity testing of daily (24 h) urinary fluoride excretion: urinary flow rate, urine volume, creatinine, other or not reported
- Method of fluoride analysis: fluoride ion-selective-electrode, gas chromatography, spectrophotometry, titration, ion-exchange chromatography, other or not reported
- Reporting of relationships between fluoride exposure and/or excretion and biomarkers (spot urine and nail clippings): yes, no

Data are described in summary tables alongside an in-depth narrative synthesis.

## Results

### Search results

**Fig. 1:**
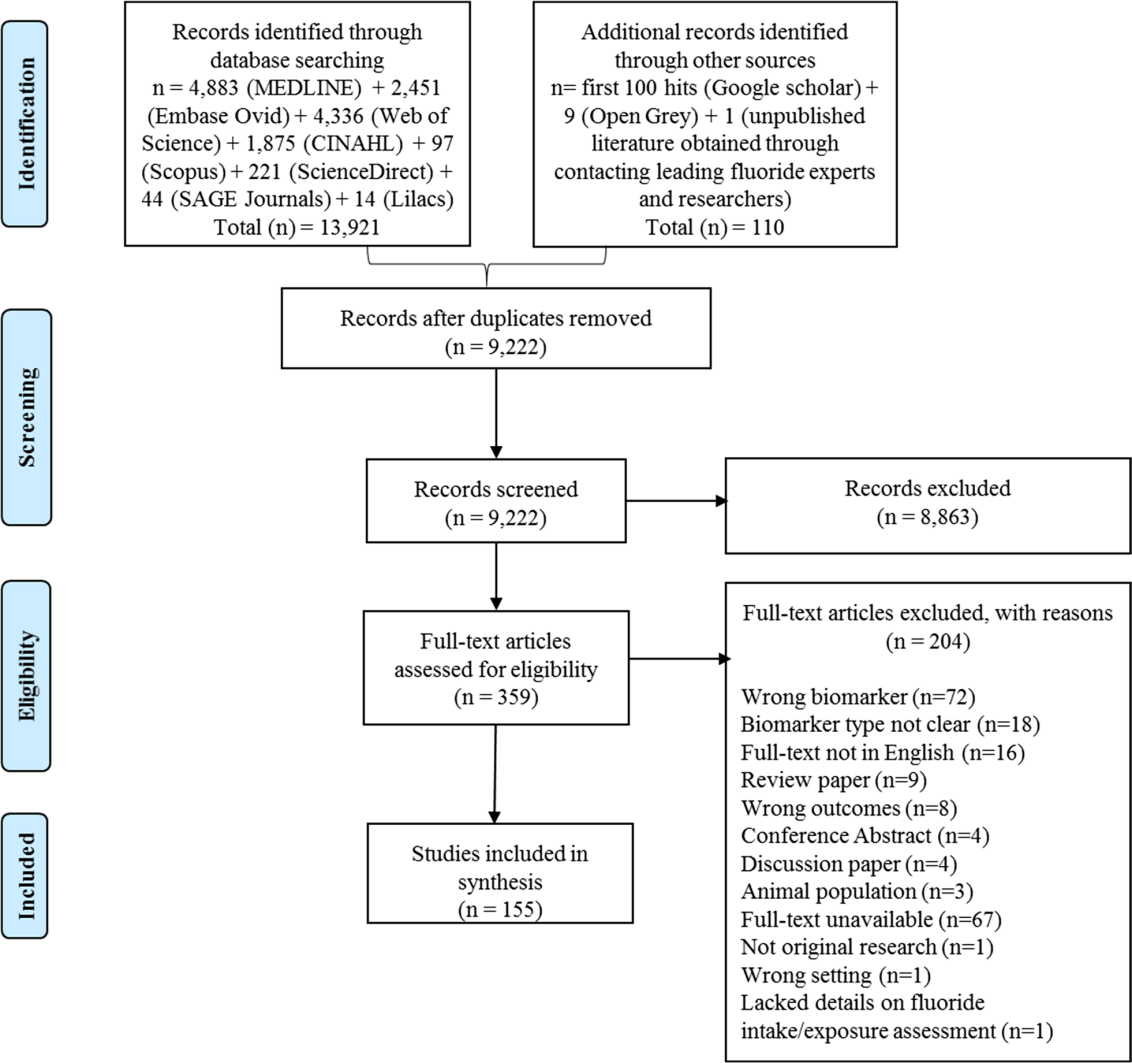
Flow diagram outlining the study selection process for inclusion in the current scoping review (adapted from Moher et al. [14])

The detailed search results are presented in Fig. 1. A total of 13,921 articles were obtained from the database searches: Medline (4883), Embase Ovid (2451), Web of Science (4336), CINAHL (1875), Scopus (97), ScienceDirect (221), SAGE Journals (44), and the Lilacs database (14). Google Scholar and Open Grey searches yielded 109 articles, whilst 1 unpublished article was obtained through contacting leading fluoride experts and researchers for potentially relevant unpublished papers. Following removal of duplicate articles, the titles and abstracts of 9222 were screened to determine their eligibility for inclusion in the next stage (i.e., full-text screening). This resulted in the exclusion of 8863 articles, which did not meet the pre-specified inclusion criteria. Consequently, the full-texts of 359 articles were screened to assess their eligibility. At the end of the full-text screening stage, 204 articles were excluded for several reasons (Fig. 1), whereas 155 studies qualified for inclusion in this review. A summary of included studies, including lists of all the 155 citations as well as the characteristics of included studies, is presented as Additional file 2.

### Characteristics of studies

**Fig. 2:**
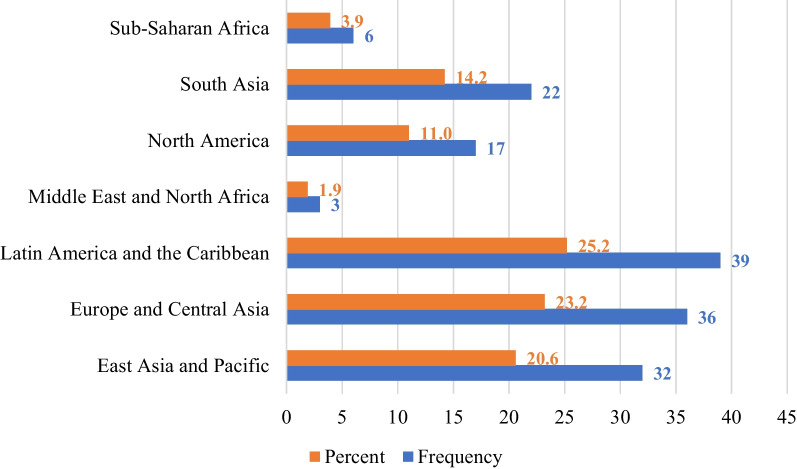
Number and percentage of studies per Continent Subregions (*Note:* Some studies were conducted in more than one country)

**Fig. 3:**
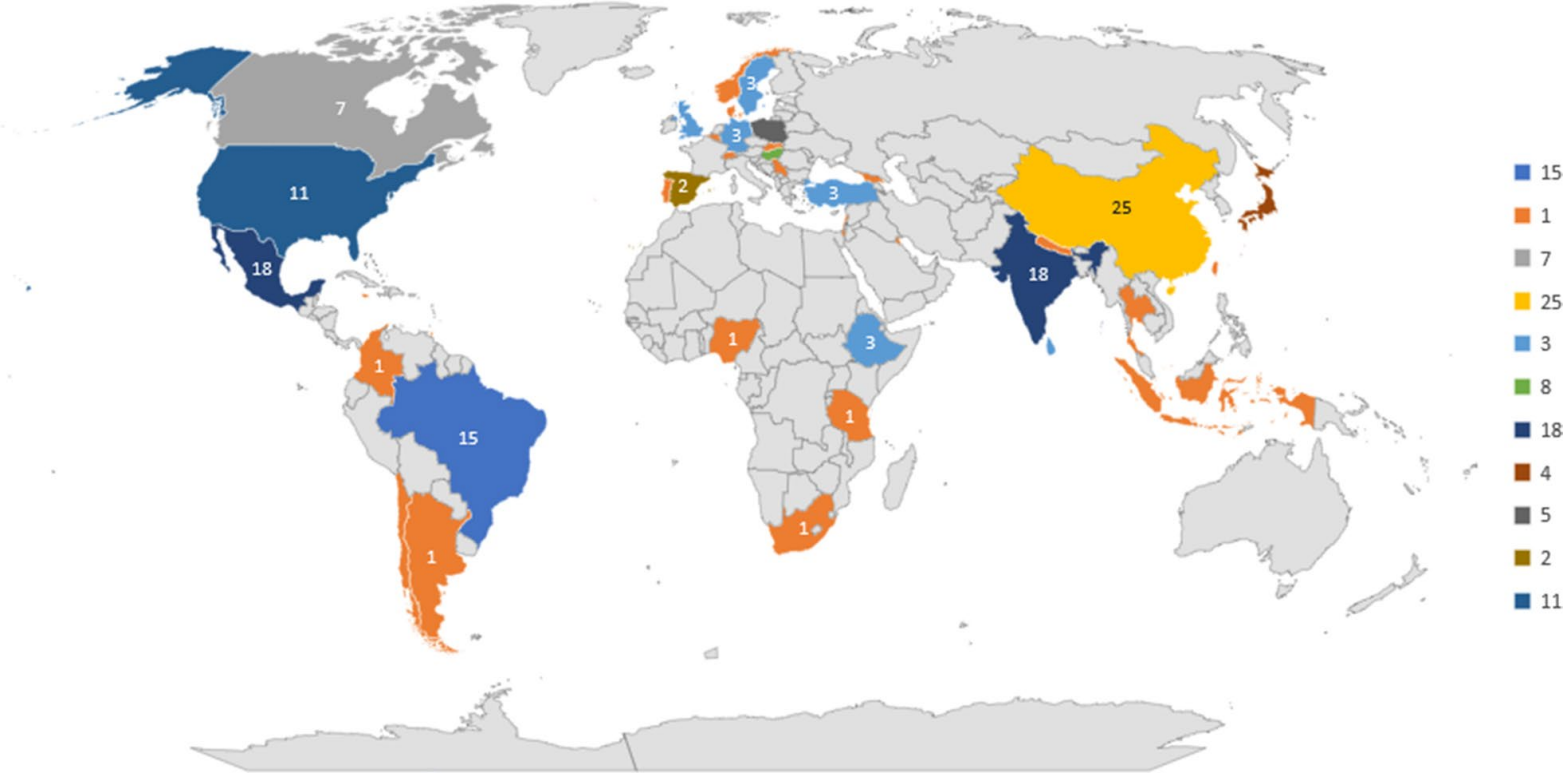
Number of included studies per country

The studies included in this review originated from 42 countries across various continents. The highest proportion of studies (25.1%) were conducted in the Latin America and the Caribbean subregion (Fig. 2). However, per country, China recorded the highest number of publications (n = 25), followed by India (n = 18), Mexico (n = 18), Brazil (n = 15), the United States of America (n = 11), Hungary (n = 8), Canada (n = 7), Poland (n = 5), and Japan (n = 4). Three studies each were conducted in the United Kingdom, Sweden, Sri Lanka, Turkey, Germany, and Ethiopia. Figure 3 presents the country distribution of included studies.

Characteristics of the included studies are presented in Table 1. Almost half the studies (47.7%) were published after 2014, and a high proportion of them (62.6%) employed a cross-sectional study design. Only 5.8% of the studies applied a randomised controlled design.

**Table 1:** Characteristics of included studies

Classification	Frequency(percentage)
*Year of publication*	
Before 2000	39 (25.2)
Between 2000 and 2013	42 (27.1)
After 2014	74 (47.7)
*Study design*	
Before and after intervention study	11 (7.1)
Case control study	5 (3.2)
Cohort study	9 (5.8)
Cross-sectional study	97 (62.6)
Longitudinal study	17 (11.0)
Non-randomised controlled study	1 (0.6)
Randomised controlled trial	9 (5.8)
Any combination of above	6 (3.9)
*Gender*	
Female	12 (7.7)
Male	9 (5.8)
Both female and male	124 (80)
Not reported	10 (6.5)
*Age*	
0–2 years	1 (0.6)
3–4 years	1 (0.6)
5–6 years	0
7–12 years	2 (1.3)
13–18 years	0
> 18 years	38 (24.5)
Any combination of above	102 (65.8)
Not reported	11 (7.1)
*Study setting*	
Childcare centres	2 (1.3)
Community settings	70 (45.2)
Hospitals	14 (9.0)
Industries	13 (8.4)
Preschools	4 (2.6)
Schools	32 (20.6)
Any combination of above	11 (7.1)
Not reported	9 (5.8)

While the studies were conducted in a range of settings, the majority were carried out in community settings (45.2%) or schools (20.6%). A high proportion (80%) of the studies included both males and females as participants, and 65.8% of the studies combined different age groups of participants. Studies conducted in individuals over 18 years old formed the highest proportion (24.5%), compared to other age groups.

## Outcome measures

### Type of biomarker

Most of the studies (83%) included in this review used spot urine samples as biomarkers for monitoring fluoride exposure. Twenty-five (16%) studies used toenails and/or fingernail clippings, whereas only 2 (1%) studies assessed both spot urine samples and toenail/fingernail clippings as biomarkers.

### Methods of assessment of fluoride intake

Table 2 presents a summary of the outcome measures related to the assessment of fluoride exposure. Most studies (66.5%) reported the mean and/or range of water fluoride concentration for the study area. Of the 103 studies that reported the water fluoride concentration, 18.4%, 35.0%, and 46.6% indicated a water fluoride level of > 1.2 mg/L, > 0.3–1.2 mg/L, and ≤ 0.3 mg/L, respectively. Over half of the studies (51.6%) reported water as the main source of fluoride exposure for the study participants, whereas a combination of diet, water, dental products, and/or medication was reported as sources of fluoride exposure in 22.6% of the included studies.

**Table 2:** Methods of assessment of fluoride exposure

	Frequency (%)
*Type of biomarker*	
Spot urine	128 (82.6)
Toenail and/or fingernail clippings	25 (16.1)
Any combination of above	2 (1.3)
*Was the water fluoride concentration of the area reported?*	
Yes	103 (66.5)
No	52 (33.5)
*If yes, what is the amount/range of fluoride level (in mg/L)*	
Up to 0.3 mg/L	19 (18.4)
> 0.3–1.2 mg/L	36 (35.0)
> 1.2 mg/L	48 (46.6)
*Sources of fluoride intake*	
Dental products	4 (2.6)
Diet	7 (4.5)
Environmental exposure	16 (10.3)
Medication	1 (0.6)
Water	80 (51.6)
Any combination of above	35 (22.6)
Not reported	12 (7.7)
*Oral hygiene assessment method*	
Applied/expectorated toothpaste	12 (7.7)
Questionnaire	9 (5.8)
Combination of above	1 (0.6)
Other: clinical examination	21 (13.5)
Not reported	112 (72.3)
*Dietary intake assessment method*	
24-h dietary recall	1 (0.6)
Diet history	3 (1.9)
Duplicate method	9 (5.8)
Food diary	2 (1.3)
Food frequency	11 (7.1)
Observed food frequency	1 (0.6)
Questionnaire	14 (9.0)
Any combination of above	4 (2.6)
Not reported	110 (71.0)
Validity of dietary intake assessment methods*	
Yes	2 (4.4)
No	43 (95.6)

*Out of the 45 studies that reported the method of assessment of dietary fluoride intake

A considerable number of the studies (72.3%) did not report their oral hygiene assessment method. Questionnaires and the applied/expectorated toothpaste methods were used by 9 and 12 studies, respectively, to assess fluoride exposure from dental products (i.e. toothpaste); whereas 21 studies reported using other methods such as clinical examination of study participants. Most studies (71%) did not report the method used in examining the dietary intake of study participants.

Only 45 (29%) of the included studies reported their dietary intake assessment approach, which consisted of seven different methods, and only 2 (4.4%) of these reported the validity of the specified dietary intake assessment method (Table 2).

### Methods of assessment of fluoride excretion

A summary of outcome measures related to fluoride excretion is presented in Table 3. Out of the 155 included studies, only 18 (11.6%) reported measuring the 24-h urinary fluoride excretion of study participants, of which 10 studies reported testing the validity of the urine sample collection. Most of the studies (n = 136, 87.7%) used the fluoride ion selective electrode to measure urinary fluoride concentration, and only 55 studies (35.5%) reported the relationship between fluoride intake and excretion.

**Table 3:** Methods of assessment of fluoride excretion

	Frequency (%)
*Was the 24-h urinary fluoride excretion reported?*	
Yes	18 (11.6)
No	137 (88.4)
*Method of validity testing*	
Creatinine ratio	3 (1.9)
Urinary flow rate	3 (1.9)
Urine volume	2 (1.3)
Urinary flow rate and creatinine	2 (1.2)
Not reported	145 (93.5)
*Method of fluoride analysis*	
Fluoride ion selective electrode	136 (87.7)
Any other method	11 (7.1)
Not reported	8 (5.2)
*Did the study report any associations between fluoride intake and excretion?*	
Yes	55 (35.5)
No	100 (64.5)

## Discussion

This scoping review aimed to ascertain the nature and extent of the available evidence on how spot urine and nail clippings are used to measure fluoride exposure, by mapping the available literature according to their study population, setting, type of study design, methodology and analytical approach. Cumulatively, the highest proportion of the studies included in this review were conducted in Latin America and the Caribbean continent sub-region. However, taken individually by country, China recorded the highest number of publications, followed by India and Mexico. This finding is consistent with a previous scoping review that focused on the use of daily urinary fluoride excretion to monitor fluoride exposure [8], as well as a previous systematic review that examined the effect of water defluoridation and improvement in fluorosis-endemic areas [15]. Over the years, countries such as China, India, and Mexico have significantly invested in fluoride research. This could be due to interest in understanding the health effects of high levels of naturally occurring fluoride in community water supplies in these countries [16, 17], which encouraged the need for continuous research on this topic.

The benefits and risks of fluoride have been of public health interest, particularly from the 1940s when the first public water fluoridation intervention for dental caries control was introduced in the United States [18]. This was later endorsed by the World Health Organisation which reinforced the use of water as the main source of fluoride to enhance oral health [18]. This high interest coupled with concerns of possible adverse health effects of fluoride have resulted in an increased number of publications in recent years [19]. Correspondingly, findings from the present scoping review indicate an increased number of studies published from the year 2000, with almost half of the studies published after 2014 (47.7%). This finding is consistent with previous reviews [8, 19, 20], and is illustrative of the on-going interest in the health effects of fluoride.

Most of the studies in this review employed a cross-sectional study design, due to the advantages of being cost-effective and less time-consuming. Cross-sectional study designs are often used to assess determinants of health, evaluate prevalence of health outcomes, and to compare differences amongst a population [21]. As such, they are appropriate for evaluating fluoride exposure.

While there has been extensive research regarding the association between fluoride exposure and related adverse health effects, studies in this area have mostly used adults as participants [22], as was also revealed in the current review. However, dental fluorosis, a well-known adverse effect of fluoride, is caused by excessive fluoride ingestion during the first 6 years of life [23]. Although diet and unintentional ingestion of fluoridated toothpaste are the main sources of fluoride exposure in children, the contribution of fluoridated toothpaste to total daily fluoride intake could be as high as 87% in children younger than 6 years of age [24]. Hence, there is a need for further studies to explore any adverse effects of fluoride exposure in children.

Fluoride biomarkers are of importance, particularly for detecting and monitoring excessive or deficient fluoride intake. In 1994, the World Health Organisation [25] established different types of biomarkers for monitoring fluoride exposure, including: (1) urine, plasma and saliva as contemporary biomarkers which assess present or very recent exposure to fluoride, (2) nails and hair as recent biomarkers, which assess recent or sub-chronic exposure to fluoride and (3) bones and teeth as historical biomarkers which assess chronic fluoride exposure.

Although daily urinary fluoride excretion has been advocated as an ideal biomarker of fluoride exposure, collection of 24-h urine samples from children, particularly those who are not toilet trained, is quite challenging. Spot urine samples and nail clippings are the most studied biomarkers due to their accessibility, processing, and storage advantages [7].

Compared to nail clippings, a large proportion (82.6%) of the studies included in this review used spot urine samples to assess fluoride exposure which could be due to them being easy to collect and, more importantly, the simplicity of the analytical methods to measure the fluoride concentration of urine.

Notwithstanding the benefits, there are potential risks with using spot urine samples to monitor fluoride exposure. The fluoride concentration of spot urine samples may be affected by several factors, including the collection time (relative to the time at which fluoride was ingested), level of hydration, as well as the length of accumulation of urine in the bladder [5]. Hence, a single spot urine sample can unlikely provide robust data on habitual exposure at the individual level. When spot urine samples are collected, it is therefore recommended to take them at several times within a day and record the collection time as well as the adjusted measure of fluoride concentration for parameters such as urinary creatinine and/or urine specific gravity which compensate for daily variation in urinary dilution of fluoride [5].

In the current review, however, the included studies did not provide sufficient information regarding the time of collection of spot urine samples, and the total number of collected spot urine samples. Additionally, biomarkers used in exposure-related health research must be validated to assure that they correctly denote the level of exposure of the researched nutrient/element. The validation criteria should include not only the analytical validity, measured according to standards, but also the biological aspects of the biomarker including its metabolism and kinetics in individuals [26]. None of the included studies in our scoping review reported biological validity of spot urine sample as a biomarker of fluoride exposure. Of the 155 studies included in this review, only 5 studies reported assessing the validity of the collected urine sample by measuring the urinary creatinine concentration. Future studies should therefore endeavour to apply rigorous methodologies in order to better understand the efficiency of spot urine samples in monitoring fluoride exposure, as well as to determine their relative usefulness.

Exposure to fluoride in water supplies has been categorised into four groups of < 0.3, 0.3– < 0.7, 0.7–1.2, and > 1.2 mg/L. From those, the 0.7–1.2 mg/L category was previously suggested as the optimal fluoride range depending on the average temperature in a community, as people in hot climates drink more water than those in moderate climates [27]. In this review, a considerable number of the included studies (51.6%) reported water as the main source of fluoride intake of study participants. Nonetheless, in a high proportion of the studies (46.6%), the water fluoride concentration of the study area was above 1.2 mg/L. Given that most of the included studies originated from China, India, and Mexico, this finding may be a result of the presence of high levels of naturally occurring fluoride in these countries.

The methods of assessing fluoride intake from diet and/or oral hygiene practice were not reported by a large proportion of the included studies (88.4%). In the few studies that reported their methods of assessment, a wide variety of methods were used. The majority of included studies reported fluoride concentration as mean values. It would be best if median and mean are reported as it is not affected by extreme outliers (e.g. unintentional ingestion of a high amount of fluoridated toothpaste by some children). Therefore, there is a need for future research to focus on developing a uniform method for an accurate assessment of fluoride exposure from diet and oral hygiene practice. Moreover, whilst many of the included studies (87.7%) used a fluoride ion selective electrode as the analytical method for measuring urinary fluoride concentration, no adequate validation information was provided.

The main limitation of studies on biomarkers of fluoride exposure is that they were not originally designed to investigate the validity of spot urine samples as a biomarker of fluoride exposure. Additionally, when interpreting urinary fluoride concentration data, it is important to be mindful of environmental and physiological factors (such as altitude, diet and age/growth rate) that could influence concentration values. None of the included studies, in our scoping review, investigated confounding variables in their analyses.

The relationship between fluoride intake and excretion was measured in 55 (out of the 155) included studies. However, a further exploration of this relationship through a systematic review/meta-analysis is recommended, considering the importance of this analysis for the understanding of the association between fluoride exposure and/or 24-h urine excretion and spot urine/nail clippings.

### Limitations of the review

The main limitation of this review is that the searches were limited to studies published in the English language. This may have led to the exclusion of potentially relevant papers published in other languages. Also, as this is a scoping review, a quality assessment of the included studies was not undertaken.

## Conclusions

This review has revealed a large variability in the way in which spot urine samples and/or nail clippings are used to measure fluoride exposure in different settings and situations. In particular, there are inconsistencies in the methodologies as well as analytical approaches used to assess exposure to fluoride. More rigorous study designs are needed to understand the use of standardised approaches to determine the suitability of spot urine samples and nail clipping as biomarkers for monitoring fluoride exposure.

## Supplementary Information

**Additional file 1.** Search strategy.

**Additional file 2.** Summary of included studies.

## Data Availability

All data generated or analysed during this study are included in this published article (and its Additional files 1, 2).
