# Supplementary material for: Mapping the evidence for monitoring fluoride exposure in community prevention programmes for oral health using nail clippings and spot urine samples: a scoping review
Source: BMC Oral Health. 2022 Dec 8;22:575. doi: 10.1186/s12903-022-02615-2 (PMC9733226; doi:10.1186/s12903-022-02615-2)
Supplement: Supplementary file 1 — Additional file 1. Search strategy. [file 12903_2022_2615_MOESM1_ESM.docx]

**Additional file 1 – Search strategy**

**Electronic Databases**

| **Database** | **MEDLINE** |
| --- | --- |
| Search query | S1 fluoride intake OR fluoride ingestion OR fluoride dose OR fluoride exposure OR fluoride content OR fluorida* OR fluoride biomarker*  S2 groundwater OR consumption OR dose* OR intake OR ingest* OR expos* OR fluorid* adj4 content OR fluoridat* OR drinking water OR exp mineral water* OR exp Water Supply  S3 diet* OR supplement* OR dentifrice* OR tablet OR salt OR milk OR dental product* OR fluoride varnish* OR mouth rinse* OR infant milk formula OR food* OR beverage  S4 S1 OR S2 OR S3  S5 spot urin* fluoride concentration* OR spot urin* fluoride excretion OR spot urin* fluoride level* OR spot urin* fluoride retention OR renal fluoride excretion OR spot urin* fluoride OR spot urin* fluoride monitor* OR spot urin* fluoride content OR fluoride balance*  S6 nail* OR nail clipping*  S7 S5 OR S6  S8 S4 AND S7 |

| **Database** | **Embase Ovid** |
| --- | --- |
| Search query | S1 (spot urin* fluoride concentration* or spot urin* fluoride excretion or spot urin* fluoride level* or spot urin* fluoride retention or renal fluoride excretion or spot urin* fluoride or spot urin* fluoride monitor* or spot urin* fluoride content or fluoride balance*).mp. [mp=title, abstract, heading word, drug trade name, original title, device manufacturer, drug manufacturer, device trade name, keyword heading word, floating subheading word, candidate term word]  S2 (nail* or nail clipping*).mp. [mp=title, abstract, heading word, drug trade name, original title, device manufacturer, drug manufacturer, device trade name, keyword heading word, floating subheading word, candidate term word]  S3 1 or 2  S4 (fluoride intake or fluoride ingestion or fluoride dose or fluoride exposure or fluoride content or fluorida* or fluoride biomarker*).mp. [mp=title, abstract, heading word, drug trade name, original title, device manufacturer, drug manufacturer, device trade name, keyword heading word, floating subheading word, candidate term word]  S5 (((groundwater or consumption or dose* or intake or ingest* or expos* or fluorid*) adj4 content) or fluoridat* or drinking water).mp. or exp mineral water*/ or exp Water Supply/ [mp=title, abstract, heading word, drug trade name, original title, device manufacturer, drug manufacturer, device trade name, keyword heading word, floating subheading word, candidate term word]  S6 (diet* or supplement* or dentifrice* or tablet or salt or milk or dental product* or fluoride varnish* or mouth rinse* or infant milk formula or food* or beverage or fluoridated water* or drink*).mp. [mp=title, abstract, heading word, drug trade name, original title, device manufacturer, drug manufacturer, device trade name, keyword heading word, floating subheading word, candidate term word]  S7 4 or 5 or 6  S8 3 and 7 |

| **Database** | **Web of Science** |
| --- | --- |
| Search query | S1 fluoride intake OR fluoride ingestion OR fluoride dose OR fluoride exposure OR fluoride content OR fluorida* OR fluoride biomarker*  S2 groundwater OR consumption OR dose* OR intake OR ingest* OR expos* OR fluorid* adj4 content OR fluoridat* OR drinking water OR exp mineral water* OR exp Water Supply  S3 diet* OR supplement* OR dentifrice* OR tablet OR salt OR milk OR dental product* OR fluoride varnish* OR mouth rinse* OR infant milk formula OR food* OR beverage  S4 S3 OR S2 OR S1  S5 spot urin* fluoride concentration* OR spot urin* fluoride excretion OR spot urin* fluoride level* OR spot urin* fluoride retention OR renal fluoride excretion OR spot urin* fluoride OR spot urin* fluoride monitor* OR spot urin* fluoride content OR fluoride balance*  S6 nail* OR nail clipping*  S7 S6 OR S5  S8 7 AND S4 |

| **Database** | **CINAHL** |
| --- | --- |
| Search query | S1 nail* OR nail clipping*  S2 spot urin* fluoride concentration* OR spot urin* fluoride excretion OR spot urin* fluoride level* OR spot urin* fluoride retention OR renal fluoride excretion OR spot urin* fluoride OR spot urin* fluoride monitor* OR spot urin* fluoride content OR fluoride balance*  S3 S1 OR S2  S4 fluoride varnish* OR mouth rinse* OR infant milk formula OR food* OR beverage  S5 groundwater OR consumption OR dose* OR intake OR ingest* OR expos* OR fluorid* adj4 content OR fluoridat* OR drinking water OR exp mineral water* OR exp Water Supply  S6 fluoride intake OR fluoride ingestion OR fluoride dose OR fluoride exposure OR fluoride content OR fluorida* OR fluoride biomarker*  S7 S4 OR S5 OR S6  S8 S3 AND S7 |

| **Database** | **Scopus** |
| --- | --- |
| Search query | ( fluoride AND intake OR fluoride AND ingestion OR fluoride AND dose OR fluoride AND exposure OR fluoride AND content OR fluorida* OR fluoride AND biomarker* ) OR ( groundwater OR consumption OR dose* OR intake OR ingest* OR expos* OR fluorid* AND adj4 AND content OR fluoridat* OR drinking AND water OR exp AND mineral AND water* OR exp AND water AND supply ) OR ( diet* OR supplement* OR dentifrice* OR tablet OR salt OR milk OR dental AND product* OR fluoride AND varnish* OR mouth AND rinse* OR infant AND milk AND formula OR food* OR beverage ) AND ( spot AND urin* AND fluoride AND concentration* OR spot AND urin* AND fluoride AND excretion OR spot AND urin* AND fluoride AND level* OR spot AND urin* AND fluoride AND retention OR renal AND fluoride AND excretion OR spot AND urin* AND fluoride OR spot AND urin* AND fluoride AND monitor* OR spot AND urin* AND fluoride AND content OR fluoride AND balance* ) OR ( nail* OR nail AND clipping* ) |

| **Database** | **ScienceDirect** |
| --- | --- |
| Search query | (fluoride exposure OR fluoride biomarker) AND oral health AND (nail clippings OR spot urine) AND (human population) |

| **Database** | **SAGE Journals** |
| --- | --- |
| Search query | [All fluoride] AND [[All exposure] OR [All fluoride]] AND [All biomarker] AND [All oral] AND [All health] AND [All nail] AND [[All clippings] OR [All spot]] AND [All urine] AND [All human population] |

**Additional sources**

| **Database** | **Google Scholar** |
| --- | --- |
| Search query | ‘fluoride exposure in community prevention programmes for oral health using nail clippings and spot urine samples’ |

| **Database** | **Open Grey** |
| --- | --- |
| Search query | [All fluoride] AND [[All exposure] OR [All fluoride]] AND [All biomarker] AND [All oral] AND [All health] AND [All nail] AND [[All clippings] OR [All spot]] AND [All urine] AND [All human population] |
